# The Role of Allostatic Load in Cognitive Impairment and Dementia in Latin America Population

**DOI:** 10.1002/alz70856_103087

**Published:** 2025-12-26

**Authors:** Nickole P. Marin‐Díaz, Matias Pizarro, Hernan Hernandez, Carolina Ochoa‐Rosales, Carolina Gonzalez‐Silva, Joaquín Migeot, Paulina Orellana, Ariel Caviedes, Hernando Santamaria‐Garcia, Andrea Slachevsky, Felipe Court, María Isabel Behrens, Diana L Matallana, Nilton Custodio, Martin Alejandro Bruno, José Alberto Avila Funes, Agustin Ibanez, Claudia Duran‐Aniotz

**Affiliations:** ^1^ Latin American Brain Health Institute (BrainLat), Universidad Adolfo Ibañez, Santiago, Chile; ^2^ Center for Social and Cognitive Neuroscience (CSCN), School of Psychology, Universidad Adolfo Ibanez., Santiago, Región Metropolitana, Chile; ^3^ Latin American Brain Health Institute (BrainLat), Universidad Adolfo Ibanez, Santiago, Región Metropolitana, Chile; ^4^ Center for Social and Cognitive Neuroscience (CSCN), School of Psychology, Universidad Adolfo Ibanez, Santiago, Chile., Santiago, Región Metropolitana, Chile; ^5^ Latin American Brain Health Institute (BrainLat), Universidad Adolfo Ibañez, Santiago, Region Metropolitana, Chile; ^6^ Latin American Institute for Brain Health (BrainLat), Universidad Adolfo Ibañez, Santiago, Santaigo, Chile; ^7^ Center for Social and Cognitive Neuroscience (CSCN), School of Psychology, Universidad Adolfo Ibañez, Santiago, Chile; ^8^ Pontificia Universidad Javeriana, Bogotá, Colombia; ^9^ Global Brain Health Institute (GBHI), University of California San Francisco (UCSF); & Trinity College Dublin, San Francisco, CA, USA; ^10^ Memory and Neuropsychiatric Center (CMYN) Neurology Department, Hospital del Salvador and Faculty of Medicine, University of Chile, Santiago, Region Metropolitana, Chile; ^11^ Geroscience Center for Brain Health and Metabolism (GERO), Santiago, Región Metropolitana de Santiago, Chile; ^12^ Servicio de Neurología, Departamento de Medicina, Clínica Alemana‐Universidad del Desarrollo, Santiago, Chile; ^13^ Memory and Neuropsychiatric Clinic (CMYN) Neurology Department, Hospital del Salvador and Faculty of Medicine, Universidad de Chile., Santiago, Chile; ^14^ Centro de Investigación Clínica Avanza (CICA), Hospital Clínico Universidad de Chile, Santiago, Chile; ^15^ Hospital Clínico de la Universidad de Chile, Santiago de Chile, Chile; ^16^ Clínica Alemana‐Universidad del Desarrollo, Santiago, Chile; ^17^ Pontificia Universidad Javeriana, Bogota, Cundinamarca, Colombia; ^18^ Hospital Universitario San Ignacio, Bogotá, Bogotá, Colombia; ^19^ Instituto Peruano de Neurociencias, Lima, Lima, Peru; ^20^ Instituto de Ciencias Biomédicas (ICBM) Facultad de Ciencias Médicas, Universidad Catoóica de Cuyo, San Juan, San Juan, Argentina; ^21^ Instituto Nacional de Ciencias Médicas y Nutrición Salvador Zubirán, México, DF, Mexico; ^22^ Global Brain Health Institute (GBHI), University of California San Francisco (UCSF); & Trinity College Dublin, Dublin, Ireland

## Abstract

**Background:**

Latin America (LA) experiences disparities driven by social determinants of health and low socioeconomic status, which increase the risk of dementia. These adverse factors may contribute to dementia through allostasis, the body's adaptive response to stressors. Chronic stressors can lead to allostatic overload, disrupting physiological systems and increasing the risk of diseases like dementia. While chronic stress is linked to cognitive decline, further research is needed to understand cumulative effects and develop an allostatic index (ALI) specific to the LA population.

**Method:**

We analyzed data from 8,044 participants across four LA cohorts: Chilean National Health Survey (Chile), GERO (Chile), ReDLat, and Costa Rica, including individuals with and without cognitive impairment or dementia. ALI was calculated using metabolic, cardiovascular, immune, neuroendocrine, and anthropometric biomarkers. Specific indices were developed for each cohort, considering cutoff values, percentiles, and risk quartiles. Logistic regression models examined associations between ALI and cognitive status, assessed with the Mini‐Mental State Examination (MMSE), Montreal Cognitive Assessment (MoCA), and Clinical Dementia Rating (CDR).

**Result:**

Higher ALI was associated with increased risk of cognitive impairment and dementia. In Costa Rica, ALI was significantly associated with cognitive impairment (OR = 1.048; *p* =  0.021). In the ReDLat cohort, higher ALI correlated with worse clinical cognitive status (OR = 1.068; *p* =  0.031). In the GERO Chile cohort, ALI showed significant impact (OR = 1.17; *p* =  0.04). In the CNHS, higher ALI notably increased cognitive impairment risk (OR = 7.15; *p* =  0.015).

**Conclusion:**

ALI is a relevant marker for identifying cognitive impairment and understanding its relationship with chronic stress. Monitoring biomarkers such as systolic blood pressure and waist‐to‐height ratio can improve the characterization of dementia in LA populations. These findings highlight the importance of addressing chronic stress and its biological impacts in diverse settings.

